# Gaucher Disease and Cancer: Concept and Controversy

**DOI:** 10.1155/2011/150450

**Published:** 2011-06-07

**Authors:** Francis Y. M. Choy, Tessa N. Campbell

**Affiliations:** ^1^Department of Biology, Centre for Biomedical Research, University of Victoria, P.O. Box 3020, Station CSC, Victoria, BC, Canada V8W 3N5; ^2^TNC Scientific Consulting, Calgary, AB, Canada

## Abstract

Gaucher disease is an inherited disorder caused by a deficiency in the lysosomal hydrolase glucocerebrosidase. There is a wide spectrum of clinical presentations, with the most common features being hepatosplenomegaly, skeletal disease, and cytopenia. Gaucher disease has been classified into three broad phenotypes based upon the presence or absence of neurological involvement: Type 1 (nonneuronopathic), Type 2 (acute neuronopathic), and Type 3 (subacute neuronopathic). The two main treatment options include enzyme replacement therapy and substrate reduction therapy. Recently, discussion has escalated around the association of Gaucher disease and cancer, with conflicting reports as to whether Gaucher patients have an increased risk of malignancy. In this review, we present both the concept and controversy surrounding the association of Gaucher disease with cancer.

## 1. Introduction

Gaucher disease is a panethnic autosomal recessive disorder characterized by a heterogeneous set of signs and symptoms caused by the defective hydrolysis of glucocerebroside. A deficiency in the enzyme glucocerebrosidase (glucosylceramidase, acid *β*-glucosidase) leads to the accumulation of glucocerebroside in the spleen, liver, and bone marrow. The resultant hepatosplenomegaly, haematological changes, and orthopaedic complications are the predominant symptoms [1]. Gaucher disease has been classified into three broad phenotypes. Type 1, the most common form, has no central nervous system involvement. Conversely, patients with Type 2 (acute neuronopathic disease) suffer from an aggressive form that leads to death perinatally or within the first few years of life. In Type 3 (subacute neuronopathic disease), patients show neurodegenerative symptoms but are able to survive through childhood to adulthood [2, 3].

The glucocerebrosidase gene is found on chromosome 1q21-22, consisting of 11 exons encoding a 497-amino-acid protein. A highly homologous pseudogene, located 16 kb downstream, complicates mutation analysis in this region. To date, nearly 300 mutations have been identified in Gaucher's patients, including point mutations, deletions, insertions, splice site mutations, frame-shift mutations, and recombinant alleles [4–6].

Gaucher disease is the most common lysosomal storage disorder and the first to be successfully treated by enzyme replacement therapy [7]. In 1991, alglucerase (Ceredase, Genzyme Inc.), the placental derivative of glucocerebrosidase was FDA-approved. In 1994, the human recombinant-form imiglucerase (Cerezyme, Genzyme Inc.) received FDA approval. Alglucerase is only available today for a handful of patients who are unable to tolerate imiglucerase [8]. In 2010, the FDA-approved velaglucerase alfa (VPRIV), a human fibroblast-derived glucocerebrosidase developed by Shire, Cambridge, Mass, USA for treatment of Gaucher disease [9, 10]. Taliglucerase alfa (UPLYSO), a recombinant glucocerebrosidase derived from plant cells developed by Protalix BioTherapeutics, Karmiel, Israel, is presently under FDA review [11].

Currently, the extreme expense of enzyme replacement therapy has led to a widespread search for more efficient and cost-effective methods of protein production and alternate therapies, resulting in a closer examination of glucocerebrosidase biosynthesis and current therapeutic techniques [12, 13]. Substrate reduction/inhibition therapy (miglustat, Zavesca, Actelion Pharmaceuticals) was approved by the EMA (2002) and FDA (2003) for adult patients unsuitable for enzyme replacement therapy. Eliglustat tartrate, a newer substrate reduction agent manufactured by Genzyme Inc., was reported to cause fewer side effects upon completion of a phase II clinical trial [14]. Other treatment avenues being explored are chaperone therapy, whereby the mutant lysosomal protein is stabilized for delivery to the lysosome, and gene therapy, which introduces the normal glucocerebrosidase gene into the cells of an affected individual [13].

This heightened search for more efficient and cost-effective treatments has led to greater scrutiny of the heterogeneity of Gaucher disease clinical presentation. One result has been the recent escalation of discussion regarding the possible correlation between Gaucher disease and cancer, with conflicting reports as to whether Gaucher's patients have an increased risk of malignancy. In this review, we present both the concept and the controversy concerning the association of Gaucher disease and cancer.

## 2. Gaucher Disease and Cancer: Concept

Numerous studies have reported an increased risk of cancer in Gaucher disease patients. Associated cancers include multiple myeloma (Table 1, [15–30]), chronic lymphocytic leukemia [31–34], chronic myeloid leukemia [35, 36], acute leukemia [37–39], large B-cell lymphoma [40], T-cell lymphoma [41], Hodgkin's disease [42–44], glioblastoma multiforme [45], lung cancer [46, 47], dysgerminoma [48], hepatocellular carcinoma [49, 50], and bone cancer [51–54]. Of the aforementioned malignancies, multiple myeloma has been most frequently reported (Table 1). The majority of the documented correlations have been with Type 1 (nonneuronopathic) Gaucher disease, with a cited cooccurrence of myeloma 6–50 times more often than expected [27, 28]. A recent study by Taddei et al. [29] delved further into this association by concentrating on individuals with the N370S mutation, the most common mutation in Type 1 Gaucher's patients. There was a conspicuous increase in lifetime risk of multiple myeloma in the entire cohort (RR 25), mostly confined to N370S homozygous patients. The risk of other hematologic malignancies (RR 3.45) and overall cancer risk (RR 1.80) was elevated. 

Several theories have been suggested to explain an association between Gaucher disease and cancer. One set of hypotheses focuses on the accumulation of glucocerebroside as the main culprit by impacting immune system regulation in a number of different ways. Costello et al. [55] postulated that exaggerated B-cell function may be secondary to stimulation by accumulated glucocerebroside, T-cell function dysregulation, augmented macrophage activation, and disruption of antigen presentation. Moreover, it has been suggested that progressive accumulation of glucocerebroside may trigger macrophage activation, leading to chronic stimulation of the immune system [56]. This could result in enhanced cytokine secretion and subsequent clonal B-cell expansion, setting the stage for eventual transformation [57]. In support of this, elevated levels of specific cytokines, including IL-1, IL-6, IL-8, IL-10, and TNF-*α*, have been found in Gaucher's patients [58–63]. Normal mesenchymal stem cells treated with conduritol B-epoxide, a potent inhibitor of glucocerebrosidase, showed an upregulation of an array of inflammatory mediators, including CCL2, and other differentially regulatory pathways [63]. Primary amyloidosis, a well-recognized consequence of hypergammaglobulinemia, has also been reported in Gaucher disease [64–66]. In another theory, immune system dysregulation resulting from glucocerebroside accumulation may also impact malignant development through reduced immune surveillance due to CD1d-mediated imbalances in regulatory and natural killer (NK) T cells [30, 67, 68]. The CD1d complex, through which NK T cells recognize glycolipids, has been reported to be elevated in Gaucher's patients and in control monocytes treated with a glucocerebrosidase inhibitor [69]. A resulting T-cell dysfunction may therefore prevent immunoregulation, allowing neoplastic cells to emerge [30, 56, 67]. Mistry et al. [70] reported that in the glucocerebrosidase gene-deficient mouse, there is widespread dysfunction of not only macrophages, but also thymic T cells, dendritic cells, and osteoblasts. 

Accumulated substrate is not the only player suggested in the proposed association of Gaucher disease and cancer. Attention has been directed towards the mutant glucocerebrosidase molecules themselves that fail to traffic to the lysosome due to posttranslational misfolding [71]. Retention in the endoplasmic reticulum may lead to the formation of potentially harmful protein aggregates, causing cellular toxicity by endoplasmic reticulum stress and proteosomal overload. Subsequent effects on cell signalling, survival and antigen presentation could impact on all aspects of Gaucher's pathology, adding a further level of complexity to the heterogeneity of the disease [57]. In theory, such a mechanism could operate in clinically unaffected carriers who possess a single missense mutation. Interestingly, a disproportionately large number of heterozygous Gaucher's mutations have been reported in patients with Parkinsonian syndromes [72].

## 3. Gaucher Disease and Cancer: Controversy

The concept of the correlation of Gaucher disease with an elevated incidence of malignancy has remained controversial, mainly due to the infrequency of the association. Though cooccurrence of some cancers, particularly myeloma, has been reported to be increased compared to the general population, malignancies are still uncommon events in the Gaucher's patient population as a whole [73]. Moreover, a number of studies have either reported no higher overall risk of cancer, at least during early or middle age, or in age-matched populations [27, 74]. Landgren et al. [75] reported that there was no specific increased risk for myeloma in Gaucher's patients, although the validity of their findings was challenged by Weinreb et al. [76] who noted that the entire cohort of patients in the Landgren study was identified as having Gaucher disease using the International Classification of Diseases 8th Revision code that includes other lysosomal storage diseases such as Fabry's disease, thus complicating the interpretation of the data. Recently, Rosenbloom et al. [77] took a reverse approach by examining multiple myeloma patients for the presence of glucocerebrosidase mutations. The 95 patients, ranging in age from 43–92 years, had bone marrow confirmation of the diagnosis of multiple myeloma. Only two patients carried a glucocerebrosidase mutation, both being heterozygous for the N370S mutation most commonly seen in Type 1 Gaucher disease. Therefore, in this study, there was no apparent relationship between heterozygosity for a Gaucher's mutation and the presence of multiple myeloma [77]. Overall, because the risk of cancer is ill-defined for Gaucher's patients, many physicians are reticent to alarm patients with regard to a potential malignancy risk in addition to their metabolic condition [57]. 

Another area of controversy involves the explanation of why a certain small subset of Gaucher's patients might develop cancer. One suggestion by Zimran et al. [78] was that the enzyme replacement therapy used to treat patients may contribute to a transformative environment by decreasing the levels of glucocerebroside and its potential accompanying anti-inflammatory and beneficial immunomodulatory effects. The authors postulate that oversuppression of the native circulatory levels of glucocerebroside by enzyme replacement therapy may disrupt the balance and induce the untoward cascade of comorbidities that are not seen in patients exposed to less exogenous enzyme. Therefore, it was suggested that, for Gaucher's patients with very mild features, enzyme replacement therapy, especially at high doses, may not be justified as the risk may be greater than the benefit. Not surprisingly, the implication of enzyme replacement therapy as a potential agent in the development of malignancy has ignited heated responses [30, 73], heightening the already controversial nature of the possible connection between Gaucher disease and cancer.

## 4. Perspectives

The extent of the association between Gaucher disease and cancer remains unclear, resulting in a climate of controversy. Adding to this complexity is emerging data on relationships between Gaucher disease and other comorbidities, such as autoimmune disease, osteoporosis, and Parkinson's disease [72, 79, 80]. The investigation of the underlying fundamental mechanisms behind such relationships warrants more study. A diverse array of available approaches, ranging from gene expression analysis to stem cell and animal models, makes such investigations a reality [81–85]. Moreover, greater sharing of information through specialized databases such as the International Collaborative Gaucher's Group Registry may serve to both clarify relationships and provide additional therapeutic targets and goals for Gaucher disease [86]. It should be noted that while the association of Gaucher disease with cancer needs further clarification, Gaucher's patients should, like normal control populations, be encouraged to participate in cancer prevention programs.

## Figures and Tables

**Table 1:** Examples of reported associations of Gaucher disease with multiple myeloma. *Note: the number in the bracket represents the reference number in the current Choy and Campbell publication.

Date	Title	Authors
1965	Coincidence of multiple myeloma with Gaucher disease	Pinkhas et al. [15]*
1968	Immunoglobulin anomalies in Gaucher disease: Report of 16 cases	Pratt et al. [16]
1979	Nonsecretory IgD-kappa multiple myeloma in a patient with Gaucher disease	Benjamin et al. [17]
1980	Coexistence of IgA myeloma and Gaucher disease	Ruestow et al. [18]
1982	Coexistence of Gaucher disease and multiple myeloma	Garfinkel et al. [19]
1988	Sequential appearance of breast carcinoma, multiple myeloma, and Gaucher disease	Gal et al. [20]
1991	Case report: serendipitous Gaucher disease presenting as elevated erythrocyte sedimentation rate due to monoclonal gammopathy	Liel et al. [21]
1993	Increased risk of cancer in patients with Gaucher disease	Shiran et al. [22]
1995	Complex IgA gammopathy in Gaucher disease	Shvidel et al. [23]
1997	Multiple myeloma arising from monoclonal gammopathy of undetermined significance in a patient with Gaucher disease	Brady et al. [24]
2000	Coincidence of Gaucher disease due to a 1226G/1448C mutation and of an immunoglobulin G lambda multiple myeloma with Bence-Jones proteinuria	Harder et al. [25]
2000	Uncommon combination of multiple myeloma in three patients	Mateja et al. [26]
2005	Gaucher disease and cancer incidence: a study from the Gaucher's Registry	Rosenbloom et al. [27]
2006	Increased incidence of cancer in adult Gaucher disease in Western Europe	De Fost et al. [28]
2009	The underrecognized progressive nature of N370S Gaucher disease and assessment of cancer risk in 403 patients	Taddei et al. [29]
2010	Expanding the spectrum of the association between Type 1 Gaucher disease and cancers: a series of patients with up to 3 sequential cancers of multiple types—correlation with genotype and phenotype	Lo et al. [30]

## Abbreviations

NK: Natural killer
EMA: European Medicines Agency
FDA: US Food and Drug Administration
RR: Relative risk
IL: Interleukin
TNF-*α*: Tumour necrosis factor-alpha
N370S: Substitution of serine for asparagine at amino acid 370.
